# Forecasting Carbon Price Using Double Shrinkage Methods

**DOI:** 10.3390/ijerph20021503

**Published:** 2023-01-13

**Authors:** Xiaolu Wei, Hongbing Ouyang

**Affiliations:** 1 Business School, Hubei University, Wuhan 430062, China; 2 School of Economics, Huazhong University of Science and Technology, Wuhan 430074, China

**Keywords:** carbon price forecasting, double shrinkage methods, factor screening, dimensionality reduction

## Abstract

It is commonly recognized that setting a reasonable carbon price can promote the healthy development of a carbon trading market, so it is especially important to improve the accuracy of carbon price forecasting. In this paper, we propose and evaluate a hybrid carbon price prediction model based on so-called double shrinkage methods, which combines factor screening, dimensionality reduction, and model prediction. In order to verify the effectiveness and superiority of the proposed model, this paper takes data from the Guangdong carbon trading market for empirical analysis. The sample interval is from 5 August 2013 to 25 March 2022. Based on the results of the empirical analysis, several main findings can be summarized. First, the double shrinkage methods proposed in this paper yield more accurate prediction results than various alternative models based on the direct application of factor screening methods or dimensionality reduction methods, when comparing R^2^, root-mean-square error (RMSE), and root absolute error (RAE). Second, LSTM-based double shrinkage methods have superior prediction performance compared to LR-based double shrinkage methods. Third, these findings are robust with the use of normalized data, different data frequencies, different carbon trading markets, and different dataset divisions. This study provides new ideas for carbon price prediction, which might have a theoretical and practical contributions to complex and non-linear time series analysis.

## 1. Introduction

Global warming is becoming one of the major environmental issues threatening the survival and development of human beings. As an effective mechanism to mitigate climate change, carbon markets have received great attention from worldwide governments and organizations. In the carbon market, carbon price forecasting is very important, which not only helps the government to make appropriate decisions and reduce investor risks, but also helps to improve carbon market construction. In light of this fact, numerous scholars have studied the predictability of carbon prices. The main research methods currently used to forecast carbon prices include traditional econometric models and machine learning models. The former is relatively simple and straightforward, mainly including linear regression models, vector autoregressive models (VARs) [1], autoregressive integrated moving average models (ARIMAs) [2], generalized autoregressive conditional heteroscedasticity models (GARCHs) [3], etc. However, these models cannot accurately capture the changes in carbon price series due to their highly nonlinear and non-stationary nature [4]. Compared with traditional econometric models, machine learning models have the advantage of high self-learning ability, high generalization ability, and associative memory, making them more suitable for fitting the non-linear relationship of a carbon price series. The common algorithms for machine learning include the backpropagation artificial neural network (BPANN) [5], the long short-term memory network (LSTM) [6], the extreme learning machine (ELM) [7], the support vector machine (SVM) [8], etc. However, the models mentioned above mainly use the information derived from the carbon price series to forecast the carbon price in the future. In light of this, some scholars have studied the influence of other factors on carbon price changes when predicting carbon prices through machine learning models. For example, Huang and He [9] further improved forecasting accuracy by investigating the effects of structured data and unstructured data on carbon prices.

In this paper, we improve the prediction accuracy of the carbon price by proposing and analyzing a double shrinkage approach to extract useful information from the potential influencing factors. First, we propose to apply the least absolute shrinkage operator (LASSO), ElasticNet shrinkage (EN), or random forest (RF) approach to select the relevant factors that contain useful information about the carbon price. Then, we apply the principal component analysis (PCA), sparse principal component analysis (s-PCA), or partial least squares method (PLS) to the selected influencing factors, in order to reduce their dimensionality and estimate latent factors of carbon prices. Finally, we use these estimated latent factors to predict carbon prices based on either linear regression models (LRs) or long short-term memory network models (LSTMs). In the first step, we shrink the set of factors that influence carbon prices by removing those factors that are not related to the change in carbon prices. In the second step, we further shrink the set of influencing factors chosen in the first step. It is in this sense that our approach can be called “double shrinkage”, which may be applied in the prediction of complex and non-linear time series in asset management, investment decision, and risk assessment.

One might argue that it is not necessary to use this method because a simpler method may have similar predictive power. However, our empirical results show that the double shrinkage method proposed in this paper yields higher prediction performance (higher R^2^, lower RMSE, and RAE) than many simpler variants of our method, including the following alternatives: (1) an LR model or LSTM model, which utilizes the raw information of the influencing factors; (2) an LR model or LSTM model, which includes influencing factors selected by LASSO, EN, or RF methods; (3) an LR model or LSTM model, which includes latent factors estimated using PCA, s-PCA, or PLS methods; and (4) an LR model or LSTM model, which includes latent factors estimated using the double shrinkage method discussed above. This may be due to the fact that the double shrinkage method discards irrelevant information while retaining relevant information about the carbon price, resulting in higher prediction accuracy.

The rest of this paper is organized as follows. Section 2 introduces the framework of our carbon price forecasting model, as well as the theories and algorithms involved. Section 3 describes our experimental setup, including details of the datasets and evaluation metrics, as well as a description of all forecasting models. Section 4 discusses the results of the empirical analysis and robustness tests. The conclusions are presented in Section 5.

## 2. The Proposed Model and Related Methods

### 2.1. Construction of the Proposed Model

**Figure 1:**
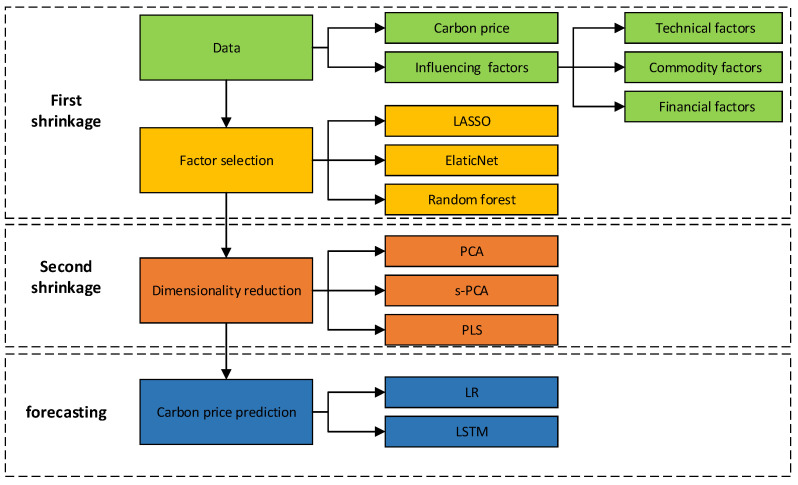
The framework of the proposed model.

The framework of the carbon prediction model based on the double shrinkage methods is presented in Figure 1. The prediction procedures of the method are as follows:

1. Data collection and preprocessing, which is represented by green in Figure 1. We collect and preprocess datasets related to carbon prices and their influencing factors; influencing factors consist of technical factors, commodity factors, and financial factors.
2. Factor selection, which is represented by yellow in Figure 1. The LASSO, EN, and RF are used to select influencing factors that may contain any useful information related to carbon prices, respectively.
3. Dimensionality reduction, which is represented by orange in Figure 1. The PCA, s-PCA, and PLS are used to remove irrelevant information contained within particular selected influencing factors, respectively.
4. Final prediction, which is represented by blue in Figure 1. Based on the estimated latent factors of carbon prices, the LR and LSTM are used to predict carbon prices, separately.

### 2.2. Related Methods

#### 2.2.1. Factor Selection Methods

In this paper, we utilize the double shrinkage methodology to construct a hybrid carbon price forecasting model, which may improve the accuracy of carbon price prediction by extracting useful information.

In the first step, we attempt to select a subset of influencing factors that are more relevant to carbon prices through factor selection methods. In this paper, we apply LASSO, ElasticNet, and random forests on the influencing factors, respectively. By removing irrelevant factors that are not crucial, the prediction model will be enhanced.

(1) LASSO

The LASSO is a regularized technique for simultaneous estimation and variable selection [10]. Imposing a penalty on the coefficients in the model, the LASSO shrinks the coefficients of irrelevant variables in the regression model to zero to constitute the subset of variables selected with non-zero coefficients.

Considering the regression of Y on $X_{i}$, the LASSO estimation is defined as:

(1) $$Q_{\mathrm{LASSO}}=\mathrm{argmin}\left\{{\left|Y-\sum_{i=1}^{n}X_{i}B_{i}\right|}^{2}+\lambda \sum_{i=1}^{n}\left|B_{j}\right|\right\}$$

where λ is a nonnegative regularization parameter, determined by cross-validation [11]. The second term is the so-called $\ell_{1}$-norm penalty, which is crucial for variable selection. Owing to the nature of the $\ell_{1}$-norm penalty, the LASSO performs both continuous shrinkage and automatic variable selection simultaneously. As λ increases, the coefficients continuously shrink toward 0 to improve the prediction accuracy [12]. In this paper, λ is chosen among [0.01, 0.05, 0.1, 0.2, 0.3, 0.4, 0.5, 1].

The above approach adds a penalty on the absolute value of the parameters to the least squares objective function, which ensures that many of the coefficients will be set to zero and thus variable selection is performed. This is an attractive feature that helps to make the results of a high-dimensional analysis interpretable. Due to this feature, the LASSO and its many extensions are now standard tools for high-dimensional analysis.

Although the LASSO has gained a high degree of success in many situations, it usually either includes a number of inactive predictors to reduce the estimation bias or over-shrinks the parameters of the correct predictors to produce a model with the correct size. These drawbacks are partially addressed by adaptive LASSO, which extends the LASSO by allowing different penalization parameters for different regression coefficients.

(2) EN

Considering an orthogonal design model [13], the LASSO shows a conflict between optimal prediction and consistent variable selection due to noisy features. Hence, Zou, and Hastie [14] proposed a new regularization technique called the elastic net (ElasticNet). The ElasticNet is a regularized least squares regression method that has been widely used in learning and variable selection. The ElasticNet penalty is a compromise between the LASSO penalty and the ridge penalty; thus, it achieves both variable selection and grouping effect. Specifically, the ElasticNet regularization linearly combines an $\ell_{1}$ penalty term (such as the LASSO) and an $\ell_{2}$ penalty term (such as ridge regression). The $\ell_{1}$ penalty term enforces sparsity of the ElasticNet estimator, whereas the $\ell_{2}$ penalty term ensures democracy among groups of correlated variables; thus, the ElasticNet estimation can be defined as:

(2) $$Q_{\mathrm{EN}}=\mathrm{argmin}\left\{{\left|Y-\overset{n}{\underset{i=1}{\sum }}X_{i}B_{i}\right|}^{2}+\lambda \overset{n}{\underset{i\;=1}{\sum }}\left(\rho \left|B_{j}\right|+\frac{\left(1-\rho \right)}{2}\right)B_{j}^{2}\right\}$$

where ρ represents the ratio of two regular terms. In our experiments, we set possible ρ as a matrix [0.01, 0.1, 0.5, 0.9, 0.99], while the selection range of λ is the same as the LASSO.

Similar to the LASSO, ElasticNet simultaneously realizes automatic variable selection and continuous shrinkage. Moreover, the $\ell_{2}$-norm penalty allows ElasticNet to select groups of correlated variables, a property that is not shared by the LASSO. However, ElasticNet is computationally more expensive than the LASSO or Ridge, as the relative weight of the LASSO versus Ridge, ρ, has to be selected using cross-validation.

(3) RF

Random forest (RF) proposed by Breiman [15] is a combination of the random subspace method [16] and the bagging method. As an ideal approach for feature selection, RF outperforms the LASSO and ElasticNet in several ways. First, RF is fairly robust in the presence of relatively high amounts of missing data [17]. Meanwhile, its computation time is modest even for very large datasets [18].

Specifically, RF first constructs multiple samples by randomly sampling data from the original samples using the bootstrap resampling technique. Then, decision trees are built and combined via the random splitting technique of nodes. Finally, the prediction results are obtained by voting.

It is worth noting that about one-third of each sample in this study is not taken. These data are called out-of-bag (OOB) data, which can be used for internal error estimates. By sorting the relative importance of variables calculated by OOB errors, variables can be screened and ranked. The variable importance measure for $x_{i}$ can be calculated as follows:

(3) $$\mathrm{VI}\left(X^{i}\right)=\frac{1}{M}\sum_{m}\left(\mathrm{err}{\tilde{{\mathrm{OOB}}_{m}}}^{i}-{\mathrm{eerOOB}}_{m}\right)$$

where ${\tilde{{\mathrm{OOB}}_{m}}}^{i}$ and $\mathrm{err}{\tilde{{\mathrm{OOB}}_{m}}}^{i}$ are computed by randomly permuting the values of $X^{i}$ in ${\mathrm{OOB}}_{m}$. For a fixed number of trees, a variable with a higher importance score indicates that the variable is significant for classification.

In the RF framework, two parameters need to be defined: the number of classification trees (N) and the number of prediction variables used by each node segmentation (M). We set n as [100, 300, 500, 800, and 1000] and m as [1, 3, 6, 8, 9, and 10], respectively.

RF models can achieve high prediction accuracy by non-parametric methods based on iterative algorithms; however, this also creates the so-called “black box” problem. This means that these models cannot interpret the causal relationship between predictors and responses.

After the factor selection process, only variables with useful information are retained. These selected factors tend to potentially provide more information about the carbon prices than others in the original set, which may be helpful for carbon price forecasting.

#### 2.2.2. Dimensionality Reduction Methods

In the second step of our procedure, we further narrow down the set of variables selected in the first step. Specifically, we apply the principal component analysis (PCA), scaled PCA (sPCA), or partial least squares (PLS) for dimensionality reduction. Eventually, we are able to estimate the potentially effective factors for carbon price forecasting.

(1) PCA

The principal component analysis (PCA) is an algorithm that transforms the columns of redundant datasets into a new set of features called principal components. Principal components contain fewer variables and retain as much information about the original variable as possible.

Mathematically, the PCA model extracts diffusion indexes as linear combinations of the predictors through the following equation:

(4) $$F_{i,t}={\;\lambda }_{i}^{\prime }F_{t}^{\mathrm{PCA}}+\epsilon_{i,t}$$

where $F_{t}^{\mathrm{PCA}}$ is a K-vector (K≪N) that denotes PCA diffusion indexes extracted from selected factors, $\lambda_{i}$ is a K-dimensional parameter to be estimated, and $\epsilon_{i,t}$ is the idiosyncratic noise term.

In this way, a large chunk of information across the full dataset is effectively compressed into fewer feature columns, thus achieving dimensionality reduction. However, the PCA is an unsupervised learning technique, which means that it ignores the prediction target and may lead to unstable prediction results. In extreme cases, when factors are strong, the PCA cannot distinguish the target-relevant and irrelevant latent factors. When the factors are weak, the PCA may fail to extract the signals from a large amount of noise, resulting in biased forecasts when all factors are used [19].

(2) sPCA

The principal component analysis (PCA) is widely used in data processing and dimensionality reduction. However, the PCA suffers from the fact that each principal component is a linear combination of all the original variables; thus, it is often difficult to interpret the results. The scaled PCA (sPCA) proposed by Huang et al. [19] is a modified principal component analysis, which assigns different weights to different predictors based on their forecasting power. Statistically, compared with the traditional PCA method evaluated based on the dimensionality reduction technique of unsupervised learning, the sPCA method is a new dimension reduction technique for supervised learning, which considers more information on statistical targets. This property allows the sPCA to overcome the deficiencies of the PCA and obtain more significant predictions. Specifically, the sPCA model extracts diffusion factors in two steps. In the first step, we form a panel of scaled predictors, ($\gamma_{1}X_{i,t},\cdots ,\gamma_{N}X_{N,t}$), where the scaled coefficient $\gamma_{1}\;$is the estimated slope obtained by regressing the prediction target on each predictor:

(5) $$y_{i,t}={\;\alpha }_{i}+\gamma_{i}X_{i,t}+\varepsilon_{t+1}$$

In the second step, we apply the PCA to the scaled predictors to extract principal components as sPCA factors and use them for prediction:

(6) $$\gamma_{i}X_{i,t}={\;\lambda }_{i}^{\prime }F_{t}^{\mathrm{sPCA}}+\epsilon_{i,t}$$

where $F_{t}^{\mathrm{sPCA}}$ is a K-vector (K≪N) that denotes sPCA diffusion indexes.

Because the prediction target $y_{t+h}$ depends on the factors instead of the loadings, sthe PCA-based prediction has a large chance to outperform the PCA-based prediction, especially when all factors are used [20,21,22,23,24].

(3) PLS

Similar to the sPCA, partial least squares (PLS) is a supervised learning method that uses the prediction target to discipline its dimension reduction process [25,26,27]. This property allows PLS to exhibit strong forecasting power even when data are relatively small [27,28,29]. Specifically, PLS extract diffusion factors in two steps as well. In the first step, we extract the component from the set of influencing factors:

(7) $$t_{1}={\;E}_{0}W_{1}$$

where $t_{h}$ is the h-th component, $E_{0}$ is the normalized matrix of X, $W_{1}$ and is the first column of $E_{0}$.

In the second step, we set up a regression equation for these components and the prediction target:

(8) $${\;\hat{F}}_{0}={\;r}_{1}t_{1}+r_{2}t_{2}+\cdots +r_{h}t_{h}$$

where $F_{0}$ is the normalized matrix of y, $r_{1}={\;E}_{0}^{T}t_{1}/{\left|\left|t_{1}\right|\right|}^{2}$.

PLS make full use of all relevant information in the variables, which lead to substantially superior forecasting performance in many areas and may be suitable for carbon price forecasting. However, PLS also have the disadvantage of a complicated calculation process and multiple iterations, which may make it difficult to interpret the regression coefficients.

## 3. Experimental Setup

### 3.1. Data

#### 3.1.1. Carbon Prices

**Figure 2:**
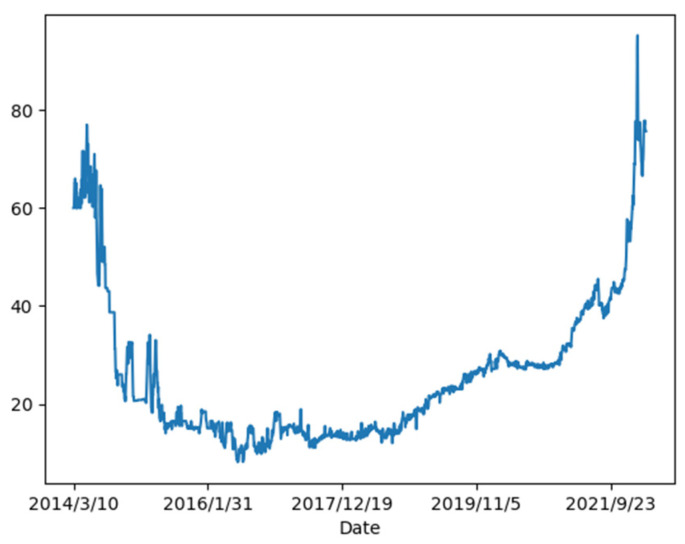
The carbon price of the Guangdong carbon trading market.

Since 2011, China has established eight carbon emissions trading pilots in Beijing, Shanghai, Tianjin, Chongqing, Hubei, Guangdong, Shenzhen, and Fujian. Among them, the carbon trading market in Guangdong had a cumulative turnover of 202.5 million tons of quotas and a cumulative turnover of CNY 4.838 billion by the end of April 2022, both of which ranked first in all carbon trading markets in China. Due to the importance of the carbon trading market in Guangdong, the carbon price of Guangdong is chosen as a detailed case for empirical analysis in this paper. Figure 2 depicts the general trend of the carbon price in Guangdong. It can be observed that the carbon price is highly nonlinear and volatile. In addition, the carbon trading market in Shanghai is used as the supplementary case to fully demonstrate the superiority and robustness of the proposed model. Shanghai is the only pilot region in China that has achieved a 100% corporate compliance clearance rate for eight consecutive years. By the end of December 2021, Shanghai’s cumulative trading volume of CCERs was 170.42 million tons, ranking first in China. Therefore, we can understand the situation of China’s carbon market well by analyzing the carbon trading markets in Guangdong and Shanghai.

We collect all the daily carbon prices of these carbon trading markets from http://k.tanjiaoyi.com/, accessed on 31 March 2022. The full sample period is from 5 August 2013 to 25 March 2022. The data with zero transaction volume are deleted from the sample. The processed sample is divided into a training set (60% of the sample), a validation set (20% of the sample), and a test set (20% of the sample). The training set is used to train the carbon price prediction models, the validation set is used to tune hyper-parameters, and the test set is used to evaluate the performance of all prediction models.

#### 3.1.2. Indicator Selection

This paper selects 71 technical indicators, 13 financial indicators, and 25 commodity indicators to forecast carbon prices. The relevant data are collected from the Wind Information, Energy Information Administration, Thomson DataStream, and Intercontinental Futures Exchange.

Specifically, the 71 technical indicators are constructed based on five popular technical rules employed by Wang et al. [30]. The details of these rules are described in Table 1.

In Table 1, $P_{t}$ denotes the carbon price for day t, Up denotes the magnitude of the upward stock price movement over k days, Down denotes the magnitude of the downward stock price movement over k days, Up+Down denotes the total magnitude of the stock price movement over the period, and ${\mathrm{MA}}_{j,t}=\left(\frac{1}{j}\right)\overset{j-1}{\underset{I\;=0}{\sum }}P_{\mathrm{tIi}}\;\left(j\;=\;s,l\right)$,$\;\mathrm{RSI}\left(k\right)=\frac{\mathrm{Up}}{\mathrm{Up}+\mathrm{Down}}\times 100$. Following Wang et al. [30], we construct five MOM indicators, twenty FR indicators, six MA indicators, twenty OSLT indicators, and twenty SR indicators, with μ = 5 and 10, k = 1, 3, 6, 9, and 12, s = 1, 3, and 6, and l = 9 and 12. Specifically, the five MOM indicators are ${\mathrm{MOM}}_{k\;=1}$, ${\mathrm{MOM}}_{k\;=3}$, ${\mathrm{MOM}}_{k\;=6}$, ${\mathrm{MOM}}_{k\;=9}$, and ${\mathrm{MOM}}_{k\;=12}$. The twenty FR indicators are ${\mathrm{FR}}_{\mu \;=5,\;k\;=1}^{\mathrm{buy}}$, ${\mathrm{FR}}_{\mu \;=5,\;k\;=3}^{\mathrm{buy}}$, ${\mathrm{FR}}_{\mu \;=5,\;k\;=6}^{\mathrm{buy}}$, ${\mathrm{FR}}_{\mu \;=5,\;k\;=9}^{\mathrm{buy}}$, ${\mathrm{FR}}_{\mu \;=5,\;k\;=12}^{\mathrm{buy}}$, ${\mathrm{FR}}_{\mu \;=10,\;k\;=1}^{\mathrm{buy}}$, ${\mathrm{FR}}_{\mu \;=10,\;k\;=3}^{\mathrm{buy}}$, ${\mathrm{FR}}_{\mu \;=10,\;k\;=6}^{\mathrm{buy}}$, ${\mathrm{FR}}_{\mu \;=10,\;k\;=9}^{\mathrm{buy}}$, ${\mathrm{FR}}_{\mu \;=10,\;k\;=12}^{\mathrm{buy}}$, ${\mathrm{FR}}_{\mu \;=5,\;k\;=1}^{\mathrm{sell}}$, ${\mathrm{FR}}_{\mu \;=5,\;k\;=3}^{\mathrm{sell}}$, ${\mathrm{FR}}_{\mu \;=5,\;k\;=6}^{\mathrm{sell}}$, ${\mathrm{FR}}_{\mu \;=5,\;k\;=9}^{\mathrm{sell}}$, ${\mathrm{FR}}_{\mu \;=5,\;k\;=12}^{\mathrm{sell}}$, ${\mathrm{FR}}_{\mu \;=10,\;k\;=1}^{\mathrm{sell}}$, ${\mathrm{FR}}_{\mu \;=10,\;k\;=3}^{\mathrm{sell}}$, ${\mathrm{FR}}_{\mu \;=10,\;k\;=6}^{\mathrm{sell}}$, ${\mathrm{FR}}_{\mu \;=10,\;k\;=9}^{\mathrm{sell}}$, ${\mathrm{FR}}_{\mu \;=10,\;k\;=12}^{\mathrm{sell}}$. The six MA indicators are ${\mathrm{MA}}_{s\;=1,l\;=9}$, ${\mathrm{MA}}_{s\;=1,l\;=12}$, ${\mathrm{MA}}_{s\;=3,l\;=9}$, ${\mathrm{MA}}_{s\;=3,l\;=12}$, ${\mathrm{MA}}_{s\;=6,l\;=9}$, and ${\mathrm{MA}}_{s\;=6,l\;=12}$. The twenty OSLT indicators are ${\mathrm{OSLT}}_{\mu \;=5,\;k\;=1}^{\mathrm{buy}}$, ${\mathrm{OSLT}\;}_{\mu \;=5,\;k\;=3}^{\mathrm{buy}}$, ${\mathrm{OSLT}\;}_{\mu \;=5,\;k\;=6}^{\mathrm{buy}}$, ${\mathrm{OSLT}\;}_{\mu \;=5,\;k\;=9}^{\mathrm{buy}}$, ${\mathrm{OSLT}\;}_{\mu \;=5,\;k\;=12}^{\mathrm{buy}}$, ${\mathrm{OSLT}\;}_{\mu \;=10,\;k\;=1}^{\mathrm{buy}}$, ${\mathrm{OSLT}\;}_{\mu \;=10,\;k\;=3}^{\mathrm{buy}}$, ${\mathrm{OSLT}\;}_{\mu \;=10,\;k\;=6}^{\mathrm{buy}}$, ${\mathrm{OSLT}\;}_{\mu \;=10,\;k\;=9}^{\mathrm{buy}}$, ${\mathrm{OSLT}\;}_{\mu \;=10,\;k\;=12}^{\mathrm{buy}}$, ${\mathrm{OSLT}\;}_{\mu \;=5,\;k\;=1}^{\mathrm{sell}}$, ${\mathrm{OSLT}\;}_{\mu \;=5,\;k\;=3}^{\mathrm{sell}}$, ${\mathrm{OSLT}\;}_{\mu \;=5,\;k\;=6}^{\mathrm{sell}}$, ${\mathrm{OSLT}\;}_{\mu \;=5,\;k\;=9}^{\mathrm{sell}}$, ${\mathrm{OSLT}\;}_{\mu \;=5,\;k\;=12}^{\mathrm{sell}}$, ${\mathrm{OSLT}\;}_{\mu \;=10,\;k\;=1}^{\mathrm{sell}}$, ${\mathrm{OSLT}\;}_{\mu \;=10,\;k\;=3}^{\mathrm{sell}}$, ${\mathrm{OSLT}\;}_{\mu \;=10,\;k\;=6}^{\mathrm{sell}}$, ${\mathrm{OSLT}\;}_{\mu \;=10,\;k\;=9}^{\mathrm{sell}}$, and ${\mathrm{OSLT}\;}_{\mu \;=10,\;k\;=12}^{\mathrm{sell}}$. The twenty SR indicators are ${\mathrm{SR}\;}_{\mu \;=5,\;k\;=1}^{\mathrm{buy}}$, ${\mathrm{SR}\;}_{\mu \;=5,\;k\;=3}^{\mathrm{buy}}$, ${\mathrm{SR}\;}_{\mu \;=5,\;k\;=6}^{\mathrm{buy}}$, ${\mathrm{SR}\;}_{\mu \;=5,\;k\;=9}^{\mathrm{buy}}$, ${\mathrm{SR}\;}_{\mu \;=5,\;k\;=12}^{\mathrm{buy}}$, ${\mathrm{SR}\;}_{\mu \;=10,\;k\;=1}^{\mathrm{buy}}$, ${\mathrm{SR}\;}_{\mu \;=10,\;k\;=3}^{\mathrm{buy}}$, ${\mathrm{SR}\;}_{\mu \;=10,\;k\;=6}^{\mathrm{buy}}$, ${\mathrm{SR}\;}_{\mu \;=10,\;k\;=9}^{\mathrm{buy}}$, ${\mathrm{SR}\;}_{\mu \;=10,\;k\;=12}^{\mathrm{buy}}$, ${\mathrm{SR}\;}_{\mu \;=5,\;k\;=1}^{\mathrm{sell}}$, ${\mathrm{SR}\;}_{\mu \;=5,\;k\;=3}^{\mathrm{sell}}$, ${\mathrm{SR}}_{\mu \;=5,\;k\;=6}^{\mathrm{sell}}$, ${\mathrm{SR}\;}_{\mu \;=5,\;k\;=9}^{\mathrm{sell}}$, ${\mathrm{SR}\;}_{\mu \;=5,\;k\;=12}^{\mathrm{sell}}$, ${\mathrm{SR}\;}_{\mu \;=10,\;k\;=1}^{\mathrm{sell}}$, ${\mathrm{SR}\;}_{\mu \;=10,\;k\;=3}^{\mathrm{sell}}$, ${\mathrm{SR}\;}_{\mu \;=10,\;k\;=6}^{\mathrm{sell}}$, ${\mathrm{SR}\;}_{\mu \;=10,\;k\;=9}^{\mathrm{sell}}$, and ${\mathrm{SR}\;}_{\mu \;=10,\;k\;=12}^{\mathrm{sell}}$.

In addition, the 13 financial indicators and 25 commodity indicators are chosen from previous literature, which shows considerable predictive power in carbon price forecasting [30,31,32,33,34,35]. The details of the financial indicators and commodity indicators are described in Table 2 and Table 3, respectively.

### 3.2. Model Accuracy Assessment

Three common evaluation metrics are selected to evaluate the performance of the prediction model. They are the coefficient of determination (R^2^), the root-mean-square error (RMSE), and the mean absolute error (MAE). Among them, the closer the value of R^2^ is to 1, while the smaller the values of RMSE and MAE, the better the prediction model performs. The three evaluation metrics are calculated as follows:

(9) $${\;R}^{2}=1-\frac{\sum_{t\;=1}^{N}{\left(y_{t}-{\;\hat{y}}_{t}\right)}^{2}}{\sum_{t\;=1}^{N}{\left(y_{t}-{\;\bar{y}}_{t}\right)}^{2}}$$

(10) $$\mathrm{RMSE}\;=\sqrt{\frac{1}{N}\overset{N}{\underset{t\;=1}{\sum }}{\left(y_{t}-{\;\hat{y}}_{t}\right)}^{2}}$$

(11) $$\mathrm{MAE}\;=\frac{1}{N}\overset{N}{\underset{t\;=1}{\sum }}\left|y_{t}-{\;\hat{y}}_{t}\right|$$

where $y_{t}$, ${\;\hat{y}}_{t}$, and ${\;\bar{y}}_{t}$ represent the true value, predicted value, and average value at time t, respectively. N is the number of samples.

In addition, we use $R_{\mathrm{OS}}^{2}$ to evaluate the out-of-sample performance of the prediction model further [36], which is calculated as follows:

(12) $$R_{\mathrm{OS}}^{2}=1-\frac{\sum_{t\;=1}^{N}{\left(y_{t}-{\;\hat{y}}_{M.t}\right)}^{2}}{\sum_{t\;=1}^{N}{\left(y_{t}-{\;\hat{y}}_{B.t}\right)}^{2}}$$

where $y_{t}$ is the true value of the prediction model, ${\;\hat{y}}_{M.t}$ is the predicted value of the prediction model, and ${\;\hat{y}}_{B.t}$ is the benchmark prediction of the historical average model. Finally, we construct Diebold–Mariano (DM) test statistics introduced by Diebold and Mariano [37] for pairwise model comparisons.

### 3.3. The Proposed Model and Comparative Methods

We examine 18 factor-augmented models associated with the proposed double shrinkage approach. They are referred to as LASSO-PCA-LR, EN-PCA-LR, RF-PCA-LR, LASSO-sPCA-LR, EN-sPCA-LR, RF-sPCA-LR, LASSO-PLS-LR, EN-PLS-LR, RF-PLS-LR, LASSO-PCA-LSTM, EN-PCA-LSTM, RF-PCA-LSTM, LASSO-sPCA- LSTM, EN-sPCA–LSTM, RF-sPCA-LSTM, LASSO-PLS-LSTM, EN-PLS-LSTM, and RF-PLS-LSTM, which are described in Table 4 under “Model Group” 6 and 7.

In addition, several groups of alternative models are included in our empirical analysis, which are also summarized in Table 4. Model Group 1, which includes LR and LSTM, is not augmented by the factor processing approach. Using this benchmark group, we can emphasize the importance of factor selection methods and dimensionality reduction methods for carbon price prediction. In Groups 2 and 3, denoted by LASSO-LR, EN-LR, RF-LR, LASSO-LSTM, EN-LSTM, and RF-LSTM, we only employ the first step of our double shrinkage approach. Namely, we apply the LASSO, EN, or RF to select a subset of factors that may contain useful information for carbon price prediction. In Groups 4 and 5, denoted by PCA-LR, sPCA-LR, PLS-LR, PCA-LSTM, sPCA-LSTM, and PLS-LSTM, we only implement the second step of our double shrinkage approach. Namely, we use PCA, sPCA, or PLS to reduce the dimensionality of the selected factors and estimate the latent factors for carbon price forecasting. In summary, there are 32 carbon price forecasting models in our empirical analysis, including 18 forecasting models based on our proposed double shrinkage approach, and 12 alternative forecasting methods, which are all described in Table 4.

## 4. Empirical Analysis

### 4.1. Forecasting Performance

This paper puts forward a hybrid carbon price prediction model based on the double shrinkage methods, which combine factor screening and dimensionality reduction to improve the accuracy of carbon price prediction. Taking Guangdong as a detailed case, the prediction results of all models are shown in Table 5. Based on all results, the analysis of each model is as follows:

1. Our double shrinkage approach results in a significant improvement in out-of-sample prediction accuracy when comparing out-of-sample R^2^ (R^2^_OOS), RMSE, and MAE. For instance, in Table 5, we see that the LASSO-sPCA-LR generates an approximately 140.47% increase in out-of-sample R^2^, an approximately 70.53% decrease in RMSE, and an approximately 61.21% decrease in MAE when compared to one of our benchmark models (LR). The LASSO-sPCA-LSTM generates an approximately 169.90% increase in out-of-sample R^2^, an approximately 87.77% decrease in RMSE, and an approximately 87.99% decrease in MAE when compared to another benchmark model (LSTM). In addition, compared with carbon prediction models based on double shrinkage methods, single prediction models (LR and LSTM), and prediction models based solely on factor selection methods or the dimensionality reduction methods are very poor, indicated by negative out-of-sample R^2^ values, and large RMSE and MAE values. For instance, the out-of-sample R^2^, RMSE, and MAE values of the PCA-LR are −2.3090, 23.8043, and 18.8319, respectively. The out-of-sample R^2^, RMSE, and MAE values of the PCA-LSTM are −2.1217, 23.1345, and 15.2526, respectively.
2. Based on in-sample R^2^, we observe that the original prediction models (LR and LSTM) generally have better in-sample fit than the carbon forecasting models based on the factor selection methods (LASSO, EN, and RF) or the dimensionality reduction methods (PCA, sPCA, and PLS), with the exception of the PCA-LSTM. In particular, the decreases in in-sample R^2^ for carbon forecasting models based on the factor selection methods or the dimensionality reduction methods range from 0.01% to 99.89% when compared with the in-sample R^2^ value of the LR and LSTM. Thus, based solely on in-sample diagnostics, there are no significant gains associated with adding a single shrinkage method to the benchmark LR or LSTM models. This indicates that the single shrinkage method may not be effective when applied without the use of the double shrinkage approach proposed in this paper. Moreover, in terms of single prediction models or prediction models based on single shrinkage methods, LR-based prediction models have better in-sample fit than LSTM-based prediction models. For instance, the in-sample R^2^ of LASSO-LR is 0.9611, while the in-sample R^2^ of LASSO-LSTM is 0.0003.
3. Based on the DM test reported in Table 5, LSTM-based carbon forecasting models show superior performance than LR-based carbon forecasting models among all double shrinkage models. Here, the alternative hypothesis of the DM test is that the prediction accuracy of the model is more accurate than that of the benchmark model. The benchmark model is based on the historical average, which is a very stringent out-of-sample benchmark for analyzing model predictability, according to Welch and Goyal [38]. The results of the DM test are indicated with an asterisk. We find that the models based on the double shrinkage methods generally have smaller RMSE and MAE compared to the alternative models, with some exceptions for LR-based prediction models. Moreover, considering all models based on the double shrinkage methods, LR-based prediction models are dominated by LSTM-based prediction models at a 1% significance level. Thus, most of our proposed models appear to be adequate for carbon price prediction, especially LSTM-based prediction models.

In conclusion, the prediction results show that the carbon price forecasting model based on the double shrinkage methods proposed in this paper usually performs better among all the models, which confirms that the double shrinkage methods have effective and superior performance in carbon price forecasting. In addition, the DM test further shows that the LSTM-based carbon price forecasting model has higher stability and feasibility among the double shrinkage methods.

### 4.2. Selected Factors

**Figure 3:**
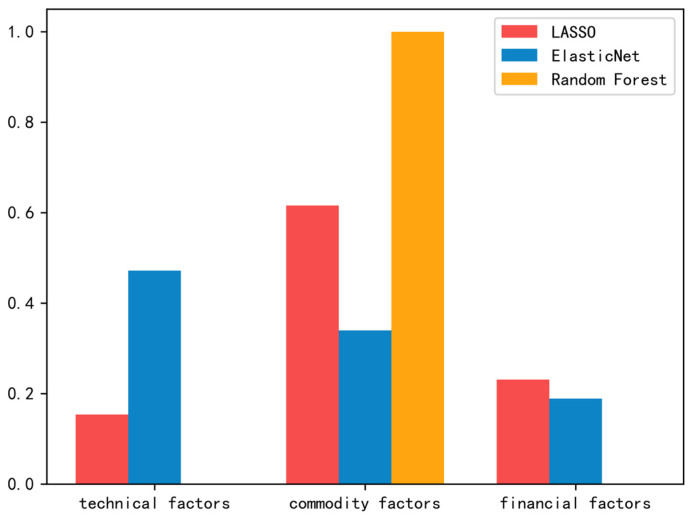
Factor selection results, by sector.

Figure 3 and Table 6, Table 7 and Table 8 summarize the results from the first step of our proposed double shrinkage methods.

Specifically, Figure 3 shows the percentages of potential factors (by sector) selected in the first step of our proposed approach. As shown in Figure 3, the commodity factors tend to be selected most frequently in the first step of our approach, except for the ElasticNet method. For the ElasticNet method, technical factors are chosen more often, with the commodity factors following closely behind. Additionally, another important and interesting point worth noting is that compared to other factor selection methods, the random forest method selects factors with a higher sector concentration, which mainly concentrates on the commodity factors.

Table 6, Table 7 and Table 8 present the most important selected factors used to construct the latent factors in the second step of our proposed approach, which are ranked by the factor importance. As shown in Table 6, Table 7 and Table 8, commodity factors show greater importance in carbon price prediction, whether using LASSO, ElasticNet, or random forest. In addition, there are significant differences among the factor selection methods in terms of factor importance. Specifically, for the LASSO method, commodity factors, financial factors, and technical factors all emerge as the most important factors in carbon price forecasting, as shown in Table 6. In contrast, Table 8 shows that for the random forest method, only one commodity factor shows very high significance in carbon price forecasting (far above 0.5).

### 4.3. Robustness Checks

#### 4.3.1. Normalization of Carbon Data

We replicate all of our experiments using normalized data. Here, the MinMax method is used for normalization. Table 9 shows the prediction results using this experimental setup, which are similar to those reported in Table 5. In Table 9, we note the following. First, and most important, the out-of-sample R^2^ values for LSTM-based double shrinkage methods are generally much higher than that of LR-based double shrinkage methods and other benchmark methods, with the exception of the LASSO-PLS-LR. The out-of-sample R^2^ value for the LASSO-PLS-LR is 0.9584. We also observe that most LR-based double shrinkage methods and other benchmark methods have poor prediction performance, indicated by negative out-of-sample R^2^ values, and large RMSE and MAE. For instance, the out-of-sample R^2^ values for LR, RF-LR PLS-LR and RF-PLS-LR are −1.1781, −1.8576, −0.3683, and −0.7406, respectively. These findings indicate that the superior performance of our proposed double shrinkage approach is largely preserved when normalization is taken, especially for LSTM-based double shrinkage methods. Second, LR and LSTM generally have a better in-sample fit than other single shrinkage methods, except for the LASSO-LSTM and PCA-LSTM. Specifically, the in-sample R^2^ value of the LSTM is 0.0182, while that of LASSO-LSTM and PCA-LSTM are 0.4432 and 0.7888, respectively. Moreover, LR-based single shrinkage methods generally have a better in-sample fit than LSTM-based single shrinkage methods, with the exception of PCA-LR and sPCA-LR. Specifically, the in-sample R^2^ values of PCA-LR and sPCA-LR are 0.0086 and 0.0106, while that of PCA-LSTM and sPCA-LSTM are 0.7888 and 0.0206, respectively.

#### 4.3.2. Different Data Frequencies

We also carried out experiments using monthly data. The results are presented in Table 10. Again, we see that LSTM-based double shrinkage methods, which are described in Group 7, generally yield larger out-of-sample R^2^, and smaller RMSE and MAE than that of LR-based double shrinkage methods and all benchmark methods. This suggests that LSTM-based double shrinkage methods are still superior, while the performances of LR-based double shrinkage methods become worse, as data frequency decreases. Additionally, all double shrinkage methods using monthly data have much worse performances than those using daily data, including LSTM-based double shrinkage methods and LR-based double shrinkage methods. We conjecture that this is because some volatile components of carbon prices become more difficult to be excluded at lower frequencies, leading to a reduction in the prediction accuracy of the double shrinkage methods. However, it should be stressed again that LSTM-based double shrinkage methods still perform very well, at the monthly frequency considered in this paper.

#### 4.3.3. Different Data Sources

To further demonstrate the results of the above analysis, this section also uses the carbon trading market in Shanghai as a supplementary case study. The prediction results of each model in Shanghai are shown in Table 11. As shown in Table 11, LSTM-based double shrinkage methods exhibit stronger prediction performance than LR-based double shrinkage methods and all benchmark methods, indicated by positive and larger out-of-sample R^2^, and smaller RMSE and MAE. This result shows that LSTM-based double shrinkage methods still have advantages in carbon price prediction, while the performance of LR-based double shrinkage methods deteriorates with the carbon trading pilot. Moreover, LR has a better in-sample fit than other LR-based single shrinkage methods, while the situation between the LSTM and LSTM-based single shrinkage methods is unclear. For instance, the in-sample R^2^ value of LR is 0.9151, while that of PCA-LR, sPCA-LR, and PLS-LR are 0.1723, 0.0553, and 0.3557, respectively. The in-sample R^2^ value of the LSTM is 0.0070, while that of PCA-LSTM, sPCA-LSTM, and PLS-LSTM are 0. 0725, 0.0642, and 0. 955, respectively. These findings are largely consistent with that of the carbon trading markets in Guangdong.

#### 4.3.4. Different Dataset Divisions

Finally, we replicate our experiments using different dataset divisions. Specifically, we divide our dataset into a training set (80% of the sample), a validation set (10% of the sample), and a test set (10% of the sample). The prediction results are gathered in Table 12. Inspection of the prediction results in this table shows that the double shrinkage methods proposed in this paper still generally outperform other benchmark methods, as evidenced by larger out-of-sample R^2^, and smaller RMSE and MAE. The only two exceptions are the LASSO-PCA-LR and EN-PCA-LR with R^2^ values of −0.9722 and −3.3258, respectively. In addition, LSTM-based double shrinkage methods are still superior to LR-based double shrinkage methods, except for the LASSO-sPCA-LR. Specifically, the R^2^, RMSE, and MAE values of the LASSO-sPCA-LR are 0.8671, 5.1716, and 4.3180, respectively, while those of the LASSO-sPCA-LSTM are 0.7636, 6.9061, and 4.3068, respectively. These results are consistent with the prediction results using 60% of the sample as the training set, indicating again the superiority of our proposed double shrinkage methods, especially LSTM-based double shrinkage methods.

### 4.4. Main Contributions and Innovations

The main contributions and innovations of this paper include the following:

(1) This paper proposes a hybrid carbon price prediction model based on the double shrinkage methods, which consist of three steps. First, the potential influencing factors of carbon prices are selected by the factor screening methods. After that, the dimensionality of the selected influencing factors is reduced by the dimensionality reduction method to estimate the latent factors of carbon prices. Finally, the carbon prices are predicted using the latent factors estimated in the previous step. The hybrid carbon prediction model proposed in this paper not only improves the prediction accuracy of the carbon price forecasting model, but also provides a new idea in the field of carbon price forecasting.
(2) In this paper, the double shrinkage methods are regarded as new keys to improving the prediction accuracy of carbon prices. By combining factor screening methods such as the LASSO, EN, and RF with factor dimensionality reduction methods such as the PCA, s-PCA, and PLS, the potential influencing factors of carbon prices are preprocessed and the latent factors of carbon prices are obtained, which are conducive to enhance the prediction accuracy of carbon price prediction model. The study results provide sufficient evidence that the use of the double shrinkage methods leads to an improvement in prediction accuracy compared to other simpler variants of our methods.
(3) In order to explore the superiority of the double shrinkage methods proposed in this paper, both linear and nonlinear models are considered to predict carbon prices. Specifically, this paper innovatively introduces the LR model and LSTM model into the field of carbon price prediction, making important theoretical and practical contributions to the literature in this area. By using the LR model and the LSTM model to predict carbon prices, the superiority of the double shrinkage methods is verified. Moreover, our empirical results fully reflect the advantages of the double shrinkage methods using LSTM, a finding that provides new insights for carbon price forecasting.

## 5. Conclusions

This paper proposes a novel carbon price forecasting model based on the double shrinkage methodology, which is composed of factor selection, dimensionality reduction, and model prediction. Taking the carbon market in Guangdong as an example, we find that the double shrinkage method greatly improves the out-of-sample forecasting accuracy of the carbon price forecasting models, as measured by the out-of-sample R^2^, root-mean-square error (RMSE), and mean absolute error (MAE). Additionally, LSTM-based double shrinkage methods always show better prediction performance than LR-based double shrinkage methods when predicting carbon prices, as indicated by higher R^2^, lower RMSE, lower MAE, and higher stability. These findings are robust to the use of original or normalized data in model specification, as well as the use of different data frequencies, different data sources, and different dataset divisions.

Although the carbon price forecasting models proposed in this paper show superior predictive performance, there are still limitations. First, this paper only uses some traditional factor selection methods (LASSO, ElasticNet, and RF) and dimensionality reduction methods (PCA, sPCA, and PLS) to construct a double shrinkage procedure. In future research, the applicability of other shrinkage methods can be further explored. Second, this paper only employs linear regression (LR) and the LSTM to predict carbon prices; other cutting-edge prediction methods can be considered in the future. Third, we could construct investment portfolios to assess whether the proposed carbon price forecasting models can be translated into profitable investments, in a real-time trading context.

## Figures and Tables

**Table 1:** Description ff technical indicators.

Number	Technical Rule	Abbreviation	Equation
1	Momentum Rule	MOM	$S_{t,\mathrm{MOM}}=\{\begin{array}{c} 1,\;\mathrm{if}\;P_{t}\geq P_{t-k}\\ 0,\;\mathrm{if}\;P_{t}<P_{t-k} \end{array}$
2	Filtering Rule	FR	$S_{t,\mathrm{FR}}^{\mathrm{buy}}=\{\begin{array}{c} 1,\;\mathrm{if}\;P_{t}\geq \left(1+\frac{\mu }{100}\right)\times \min\left(P_{t-1},P_{t-2},\cdots ,P_{t-k}\right)\\ 0,\;\mathrm{otherwise} \end{array}$ $S_{t,\mathrm{FR}}^{\mathrm{sell}}=\{\begin{array}{c} 1,\;\mathrm{if}\;P_{t}\leq \left(1+\frac{\mu }{100}\right)\times \max\left(P_{t-1},P_{t-2},\cdots ,P_{t-k}\right)\\ 0,\;\mathrm{otherwise} \end{array}$
3	Moving Average Rule	MA	$S_{t,\mathrm{MA}}=\{\begin{array}{c} 1,\;\mathrm{if}\;{\mathrm{MA}}_{s,t}\geq {\mathrm{MA}}_{l,t}\\ 0,\;\mathrm{otherwise} \end{array}$
4	Oscillator Rule	OSLT	$S_{t,\mathrm{OSLT}}^{\mathrm{buy}}=\{\begin{array}{c} 1,\;\mathrm{if}\;{\mathrm{RSI}}_{t}\leq 50+\mu \\ 0,\;\mathrm{otherwise} \end{array}$ $S_{t,\mathrm{OSLT}}^{\mathrm{sell}}=\{\begin{array}{c} 1,\;\mathrm{if}\;{\mathrm{RSI}}_{t}\geq 50+\mu \\ 0,\;\mathrm{otherwise} \end{array}$
5	Support/Resistance Rule	SR	$S_{t,\mathrm{SR}}^{\mathrm{buy}}=\{\begin{array}{c} 1,\;\mathrm{if}\;P_{t}\geq \left(1+\frac{\mu }{100}\right)\times \max\left(P_{t-1},P_{t-2},\cdots ,P_{t-k}\right)\\ 0,\;\mathrm{otherwise} \end{array}$ $S_{t,\mathrm{SR}}^{\mathrm{sell}}=\{\begin{array}{c} 1,\;\mathrm{if}\;P_{t}\leq \left(1+\frac{\mu }{100}\right)\times \min\left(P_{t-1},P_{t-2},\cdots ,P_{t-k}\right)\\ 0,\;\mathrm{otherwise} \end{array}$

**Table 2:** Description of financial indicators.

Number	Financial Indicators	Abbreviation	Description
1	S&P 500 index	SP500	-
2	Dow Jones Composite Index	DJ	-
3	Shanghai Composite Index	SHANGHAI COMPOSITE INDEX	-
4	Shenzhen Composite Index	SZSE COMPONENT INDEX	-
5	5-Year Bond Index Yield	BOND	-
6	AAA-Rated Corporate Bond Spreads	COPBOND SPREAD	Daily spread between AAA-rated corporate bonds and BAA-rated corporate bonds
7	Treasury Rate	TREASURY RATE	Secondary market interest rates for 3-month Treasury bills
8	Long-term Treasury Spreads	TREASURY SPREAD	Daily spreads between 1-year Treasury bills and 10-year state bonds
9	Long-term Treasury Yield	LONTREASURY YIELD	10-year Treasury rate
10	Exchange Rate (US)	USD/CNY	-
11	China Economic Policy Uncertainty Index	UNCERTAINTY	-
12	WilderHill New Energy Global Innovation Index	NEX	-
13	WilderHill Clean Energy Index	CEI	-

**Table 3:** Description of commodity indicators.

Number	Commodity Indicators	Abbreviation
1	ICE-UK natural gas continuous futures price	UKGP
2	Asia gas price	JKM
3	S&P GSCI gas oil index excess return	GGO
4	ICE-coal Rotterdam continuous futures price	GP
5	ICE-Brent crude oil continuous futures price	BOP
6	S&P GSCI crude oil index excess return	GCO
7	EUA price	EUA
8	China Electricity Price index	CEP
9	S&P GSCI non-energy commodity indexes	Including GGOL, GSIL, GALU, GCOP, GLEA, GNIC, GZIN, GCOC, GCOF, GCOR, GCOT, GSOY, GSUG, GWHE, GFC, GLH, AND GLC

**Table 4:** Description of carbon price forecasting models.

Group	Model	Description
1	LR LSTM	Prediction models with only raw factor information
2	LASSO-LR	Linear models augmented by factors selected using the LASSO method
	EN-LR	Linear models augmented by factors selected using the EN method
	RF-LR	Linear models augmented by factors selected using the RF method
3	LASSO-LSTM	Nonlinear models augmented by factors selected using the LASSO method
	EN-LSTM	Nonlinear models augmented by factors selected using the EN method
	RF-LSTM	Nonlinear models augmented by factors selected using the RF method
4	PCA-LR	Linear models augmented by the latent factors estimated using the PCA method
	sPCA-LR	Linear models augmented by the latent factors estimated using the s-PCA method
	PLS-LR	Linear models augmented by the latent factors estimated using the PLS method
5	PCA-LSTM	Nonlinear models augmented by the latent factors estimated using the PCA method
	sPCA-LSTM	Nonlinear models augmented by the latent factors estimated using the s-PCA method
	PLS-LSTM	Nonlinear models augmented by the latent factors estimated using the PLS method
6	LASSO-PCA-LR	Linear models augmented by the latent factors estimated using LASSO-selected factors and the PCA method
	EN-PCA-LR	Linear models augmented by the latent factors estimated using EN-selected factors and the PCA method
	RF-PCA-LR	Linear models augmented by the latent factors estimated using RF-selected factors and the PCA method
	LASSO-sPCA-LR	Linear models augmented by the latent factors estimated using LASSO-selected factors and the s-PCA method
	EN-sPCA-LR	Linear models augmented by the latent factors estimated using EN-selected factors and the s-PCA method
	RF-sPCA-LR	Linear models augmented by the latent factors estimated using RF-selected factors and the s-PCA method
	LASSO-PLS-LR	Linear models augmented by the latent factors estimated using LASSO-selected factors and the PLS method
	EN-PLS-LR	Linear models augmented by the latent factors estimated using EN-selected factors and the PLS method
	RF-PLS-LR	Linear models augmented by the latent factors estimated using RF-selected factors and the PLS method
7	LASSO-PCA-LSTM	Nonlinear models augmented by the latent factors estimated using LASSO-selected factors and the PCA method
	EN-PCA-LSTM	Nonlinear models augmented by the latent factors estimated using EN-selected factors and the PCA method
	RF-PCA-LSTM	Nonlinear models augmented by the latent factors estimated using RF-selected factors and the PCA method
	LASSO-sPCA-LSTM	Nonlinear models augmented by the latent factors estimated using LASSO-selected factors and the s-PCA method
	EN-sPCA-LSTM	Nonlinear models augmented by the latent factors estimated using EN-selected factors and the s-PCA method
	RF-sPCA-LSTM	Nonlinear models augmented by the latent factors estimated using RF-selected factors and the s-PCA method
	LASSO-PLS-LSTM	Nonlinear models augmented by the latent factors estimated using LASSO-selected factors and the PLS method
	EN-PLS-LSTM	Nonlinear models augmented by the latent factors estimated using EN-selected factors and the PLS method
	RF-PLS-LSTM	Nonlinear models augmented by the latent factors estimated using RF-selected factors and the PLS method

**Table 5:** Forecast results.

Model	R^2^_IS	R^2^_OOS	RMSE	MAE
LR	0.9611	−1.8576	22.1211 ***	14.3944 ***
LSTM	0.2813	−1.3796	20.1983 ***	15.0991 ***
LASSO-LR	0.9611	−1.8576	22.1211 ***	14.3944 ***
EN-LR	0.9611	−1.8576	22.1211 ***	14.3944 ***
RF-LR	0.9611	−1.8576	22.1211 ***	14.3944 ***
LASSO-LSTM	0.0003	−0.9249	18.1662 ***	12.7558 ***
EN-LSTM	0.0008	−0.9405	18.2396 ***	12.8149 ***
RF-LSTM	0.0003	−0.9230	18.1573 ***	12.7443 ***
PCA-LR	0.0949	−2.3090	23.8043 ***	18.8319 ***
sPCA-LR	0.0095	−1.0288	18.6389 ***	13.3949 ***
PLS-LR	0.6012	−0.3683	15.3074 ***	12.9321 ***
PCA-LSTM	0.9985	−2.1217	23.1345 ***	15.2526 ***
sPCA-LSTM	0.0174	−1.0586	18.7867 ***	13.5221 ***
PLS-LSTM	0.0115	−1.0159	18.5905 ***	13.2900 ***
LASSO-PCA-LR	0.7921	−2.1457	23.2094 ***	22.2432 ***
EN-PCA-LR	0.1583	−3.8264	28.7487 ***	23.3717 ***
RF-PCA-LR	0.6530	−1.9122	22.3313 ***	16.1361 ***
LASSO-sPCA-LR	0.0895	0.7518	6.5198 ***	5.5832 ***
EN-sPCA-LR	0.0087	−1.0069	18.5383 ***	13.2330 ***
RF-sPCA-LR	0.6327	0.4296	9.8829 ***	5.9880 ***
LASSO-PLS-LR	0.8186	0.1408	12.1300 ***	10.3330 ***
EN-PLS-LR	0.6671	−0.0103	13.1529 ***	10.8795 ***
RF-PLS-LR	0.6475	−0.6443	16.7803 ***	12.1148 ***
LASSO-PCA-LSTM	0.9918	0.9106	3.9159 ***	3.2590 ***
EN-PCA-LSTM	0.9971	0.9397	3.2161 ***	2.1659 ***
RF-PCA-LSTM	0.9989	0.8605	4.8900 ***	2.4780 ***
LASSO-sPCA-LSTM	0.9970	0.9644	2.4709 ***	1.8130 ***
EN-sPCA-LSTM	0.9934	0.935	3.3388 ***	2.1705 ***
RF-sPCA-LSTM	0.9922	0.7853	6.0667 ***	2.7855 ***
LASSO-PLS-LSTM	0.9919	0.9552	2.7724 ***	1.7157 ***
EN-PLS-LSTM	0.9933	0.9343	3.3555 ***	2.5518 ***
RF-PLS-LSTM	0.9941	0.9425	3.1398 ***	1.4301 ***

Note: *, **, and *** represent statistical significance at the 10%, 5%, and 1% levels, respectively.

**Table 6:** Description of the most important selected factors, using LASSO.

Factor	Sector	Importance
GGOL	Commodity factor	5.7600
EUA	Commodity factor	5.2540
GCO	Commodity factor	4.3160
GWHE	Commodity factor	3.7520
GGP	Commodity factor	2.4190
SHANGHAI	Financial factor	1.7910
BOP	Commodity factor	1.4640
GCOR	Commodity factor	1.2700
USD/CNY	Financial factor	1.2570
TREASURY RATE	Financial factor	1.0360
$S_{\mathrm{FR}}^{\mathrm{sell}}$(k = 12,η = 5)	Technical factor	0.5930
GALU	Commodity factor	0.1990
$S_{\mathrm{FR}}^{\mathrm{buy}}$(k = 9,η = 10)	Technical factor	0.1000

**Table 7:** Description of the most important selected factors, using ElasticNet.

Factor	Sector	Importance
EUA	Commodity factor	3.8300
GGOL	Commodity factor	3.4500
CEP	Commodity factor	3.3800
BOP	Commodity factor	2.6310
GWHE	Commodity factor	2.4450
GCO	Commodity factor	2.1310
USD/CNY	Financial factor	1.8330
GCOR	Commodity factor	1.6020
GNIC	Commodity factor	1.5330
GGP	Commodity factor	1.1890

**Table 8:** Description of the most important selected factors, using random forest.

Factor	Sector	Importance
GNIC	Commodity factor	0.9960
CEI	Financial factor	0.0040

**Table 9:** Forecast results using normalized data.

Model	R^2^_IS	R^2^_OOS	RMSE	MAE
LR	0.9611	−1.1781	19.3240 ***	14.0085 ***
LSTM	0.0182	−0.9285	18.1833 ***	12.7778 ***
LASSO-LR	0.9611	−1.8576	22.1211 ***	14.3944 ***
EN-LR	0.9611	−1.8576	22.1211 ***	14.3944 ***
RF-LR	0.9611	−1.8576	22.1211 ***	14.3944 ***
LASSO-LSTM	0.4432	−1.2709	19.7314 ***	14.7666 ***
EN-LSTM	0.0024	−0.9556	18.3105 ***	12.7730 ***
RF-LSTM	0.0001	−0.9284	18.1827 ***	12.7771 ***
PCA-LR	0.0086	−1.0283	18.6367 ***	13.4345 ***
sPCA-LR	0.0106	−1.0339	18.6625 ***	13.4205 ***
PLS-LR	0.6012	−0.3683	15.3074 ***	12.9321 ***
PCA-LSTM	0.7888	−0.1331	13.9299 ***	12.3688 *
sPCA-LSTM	0.0206	−1.0600	18.7931 ***	13.5422 ***
PLS-LSTM	0.0186	−1.0637	18.8098 ***	13.5756 ***
LASSO-PCA-LR	0.0107	−1.0811	18.8778 ***	13.6999 ***
EN-PCA-LR	0.0002	−0.9296	18.1775 ***	12.7151 ***
RF-PCA-LR	0.7026	−3.5688	27.9708 ***	23.6114 ***
LASSO-sPCA-LR	0.0058	−1.0064	18.5359 ***	13.2752 ***
EN-sPCA-LR	0.0084	−1.0240	18.6171 ***	13.3718 ***
RF-sPCA-LR	0.0098	−1.0529	18.7603 ***	13.5782 ***
LASSO-PLS-LR	0.9925	0.9584	2.6692 ***	1.7970 ***
EN-PLS-LR	0.7228	−0.1151	13.8187 ***	12.1050 *
RF-PLS-LR	0.7507	−0.7406	17.2646 ***	13.9340 ***
LASSO-PCA-LSTM	0.993	0.9621	2.5488 ***	1.7249 ***
EN-PCA-LSTM	0.9986	0.9785	1.9220 ***	0.9886 ***
RF-PCA-LSTM	0.9939	0.9268	3.5435 ***	2.9874 ***
LASSO-sPCA-LSTM	0.5862	0.4087	10.9099 ***	6.1710 ***
EN-sPCA-LSTM	0.994	0.9082	3.9678 ***	2.6631 ***
RF-sPCA-LSTM	0.9877	0.6045	8.9322 ***	4.5289 ***
LASSO-PLS-LSTM	0.9926	0.9187	3.7325 ***	2.4460 ***
EN-PLS-LSTM	0.9926	0.919	3.7261 ***	2.4405 ***
RF-PLS-LSTM	0.9935	0.9309	3.4414 ***	2.3839 ***

Note: *, **, and *** represent statistical significance at 10%, 5%, and 1% levels, respectively.

**Table 10:** Forecast results using monthly data.

Model	R^2^_IS	R^2^_OOS	RMSE	MAE
LR	0.9998	−106.5604	127.7246 ***	100.2299 ***
LSTM	0.2496	−1.9336	21.3694 ***	17.5679 ***
LASSO-LR	0.9998	−106.5604	127.7246 ***	100.2299 ***
EN-LR	0.9998	−106.5604	127.7246 ***	100.2299 ***
RF-LR	0.9998	−106.5604	127.7246 ***	100.2299 ***
LASSO-LSTM	0.2256	−1.3354	19.0666 ***	14.8047 ***
EN-LSTM	0.5956	−1.7625	20.7367 ***	17.1611 ***
RF-LSTM	−0.0020	−0.8835	17.1226 ***	12.0430 ***
PCA-LR	0.0569	−0.9098	17.0192 ***	13.2268 ***
sPCA-LR	0.4768	−21.3069	58.1658 ***	49.9827 ***
PLS-LR	0.6787	−0.0624	12.6937 ***	10.6888 *
PCA-LSTM	0.1459	−1.7658	20.7492 ***	7.2182 ***
sPCA-LSTM	0.9371	−3.2593	25.7487 ***	20.7535 ***
PLS-LSTM	0.2858	−1.7450	20.671 ***	8.2773 ***
LASSO-PCA-LR	0.1666	−1.5844	19.7983 ***	16.4299 ***
EN-PCA-LR	0.0419	−0.8303	16.6611 ***	12.6514 ***
RF-PCA-LR	0.7375	−0.4339	14.7471 ***	12.4873 *
LASSO-sPCA-LR	0.0451	−1.0229	17.5161 ***	12.9175 ***
EN-sPCA-LR	0.0477	−1.0591	17.6722 ***	13.1878 ***
RF-sPCA-LR	0.9309	−0.0788	12.9589 ***	6.0113 *
LASSO-PLS-LR	0.7472	−0.0522	12.6328 ***	10.7025 *
EN-PLS-LR	0.6318	0.0730	11.8574 ***	9.9209 ***
RF-PLS-LR	0.7350	−0.1440	13.1725 ***	10.9729 *
LASSO-PCA-LSTM	0.9142	0.2862	10.5409 ***	8.7097 *
EN-PCA-LSTM	0.9131	0.4579	9.1858 ***	6.7665 *
RF-PCA-LSTM	0.9288	0.0396	12.2271 ***	10.4447 *
LASSO-sPCA-LSTM	0.9135	0.4666	9.1117 ***	16.5321 *
EN-sPCA-LSTM	0.9232	0.1690	11.3732 ***	16.4778 *
RF-sPCA-LSTM	0.9925	0.8094	5.7164 ***	2.7658 ***
LASSO-PLS-LSTM	0.6554	0.4490	9.7139 ***	10.3195 ***
EN-PLS-LSTM	0.9208	0.1656	11.3965 ***	8.6879 *
RF-PLS-LSTM	0.9326	0.1945	11.1972 ***	9.3815 *

Note: *, **, and *** represent statistical significance at 10%, 5%, and 1% levels, respectively.

**Table 11:** Forecast results for Shanghai.

Model	R^2^_IS	R^2^_OOS	RMSE	MAE
LR	0.9151	−113.6345	46.3574 ***	40.3219 ***
LSTM	0.0070	−7.3804	12.5412 ***	11.8109 ***
LASSO-LR	0.9151	−113.6345	46.3574 ***	40.3219 ***
EN-LR	0.9151	−113.6345	46.3574 ***	40.3219 ***
RF-LR	0.9151	−113.6345	46.3574 ***	40.3219 ***
LASSO-LSTM	0.0076	−7.3904	12.5487 ***	11.8172 ***
EN-LSTM	-0.0007	−7.5129	12.6400 ***	11.9172 ***
RF-LSTM	0.0500	−7.3255	12.5001 ***	11.7454 ***
PCA-LR	0.1723	−5.4494	10.9956 ***	9.8074 ***
sPCA-LR	0.0553	−7.9520	12.9545 ***	12.1597 ***
PLS-LR	0.3557	−2.1447	7.6781 ***	6.4188 ***
PCA-LSTM	0.0725	−7.5386	12.6590 ***	11.8781 ***
sPCA-LSTM	0.0642	−7.9157	12.9356 ***	12.0140 ***
PLS-LSTM	0.9955	−1.9432	7.4322 ***	6.5363 ***
LASSO-PCA-LR	0.1047	−9.2810	13.8828 ***	13.0996 ***
EN-PCA-LR	0.1726	−5.8431	11.3263 ***	10.2128 ***
RF-PCA-LR	0.0589	−1.3319	6.6118 ***	5.2805 ***
LASSO-sPCA-LR	0.2243	−6.7914	12.0856 ***	10.9651 ***
EN-sPCA-LR	0.0540	−7.9533	12.9555 ***	12.1673 ***
RF-sPCA-LR	0.0355	−14.3159	16.9446 ***	16.2228 ***
LASSO-PLS-LR	0.7143	−1.4267	6.7448 ***	5.0866 ***
EN-PLS-LR	0.3594	−2.0975	7.6202 ***	6.3697 ***
RF-PLS-LR	0.4758	−7.8180	12.8572 ***	9.8501 ***
LASSO-PCA-LSTM	0.9956	0.6019	2.7333 ***	1.4820 ***
EN-PCA-LSTM	0.9983	0.6304	2.6338 ***	1.4915 ***
RF-PCA-LSTM	0.9966	0.5131	3.0231 ***	2.0736 ***
LASSO-sPCA-LSTM	0.9986	0.6472	2.5734 ***	1.3672 ***
EN-sPCA-LSTM	0.9984	0.6244	2.6550 ***	1.4870 ***
RF-sPCA-LSTM	0.9986	0.6197	2.6715 ***	1.4868 ***
LASSO-PLS-LSTM	0.9992	0.5943	2.7593 ***	1.4090 ***
EN-PLS-LSTM	0.9987	0.4408	3.2397 ***	1.8249 ***
RF-PLS-LSTM	0.9977	0.6978	2.3816 ***	1.3975 ***

Note: *, **, and *** represent statistical significance at 10%, 5%, and 1% levels, respectively.

**Table 12:** Forecast results for different dataset divisions.

Model	R^2^_IS	R^2^_OOS	RMSE	MAE
LR	0.9525	−3.4916	30.0679 ***	23.1696 ***
LSTM	0.0018	−1.8725	24.0738 ***	19.4331 ***
LASSO-LR	0.9525	−3.4916	30.0679 ***	23.1696 ***
EN-LR	0.9525	−3.4916	30.0679 ***	23.1696 ***
RF-LR	0.9525	−3.4916	30.0679 ***	23.1696 ***
LASSO-LSTM	0.0007	−1.8742	24.0807 ***	19.4416 ***
EN-LSTM	0.0002	−1.8703	24.0646 ***	19.4217 ***
RF-LSTM	0.0007	−1.8778	24.0960 ***	19.4561 ***
PCA-LR	0.0327	−2.6362	27.0539 ***	22.8044 ***
sPCA-LR	0.0023	−1.9353	24.3069 ***	19.7415 ***
PLS-LR	0.9936	−0.0766	14.7380 ***	9.0126 ***
PCA-LSTM	0.0035	−1.8896	24.1454 ***	19.5212 ***
sPCA-LSTM	0.0157	−1.8800	24.1049 ***	19.3604 ***
PLS-LSTM	0.0029	−1.9345	24.3037 ***	19.7178 ***
LASSO-PCA-LR	0.3738	−0.9722	19.9242 ***	18.1494 ***
EN-PCA-LR	0.0514	−3.3258	29.5076 ***	25.2293 ***
RF-PCA-LR	0.5504	0.2802	12.0365 ***	9.6932 ***
LASSO-sPCA-LR	0.1078	0.8671	5.1716 ***	4.3180 ***
EN-sPCA-LR	0.4370	0.2115	12.5979 ***	10.8624 **
RF-sPCA-LR	0.5862	0.4087	10.9099 ***	6.1710 ***
LASSO-PLS-LR	0.5877	0.7369	7.2777 ***	5.6916 ***
EN-PLS-LR	0.5228	0.3315	11.5996 ***	9.4346 ***
RF-PLS-LR	0.5599	0.3465	11.4693 ***	9.0028 ***
LASSO-PCA-LSTM	0.9945	0.9583	2.9009 ***	1.2980 ***
EN-PCA-LSTM	0.9954	0.9331	3.6727 ***	1.8286 ***
RF-PCA-LSTM	0.9989	0.9679	2.5435 ***	1.0284 ***
LASSO-sPCA-LSTM	0.9964	0.7636	6.9061 ***	4.3068 ***
EN-sPCA-LSTM	0.9955	0.9540	3.0456 ***	1.3010 ***
RF-sPCA-LSTM	0.9877	0.6045	8.9322 ***	4.5289 ***
LASSO-PLS-LSTM	0.9973	0.9652	2.6482 ***	1.4527 ***
EN-PLS-LSTM	0.9971	0.9140	4.1648 ***	1.8921 ***
RF-PLS-LSTM	0.9989	0.9671	2.5762 ***	1.0513 ***

Note: *, **, and *** represent statistical significance at 10%, 5%, and 1% levels, respectively.

## Data Availability

The data presented in this study are available within the article.
